# Development of a Claims-Based Algorithm to Identify Patients With Agitation in Alzheimer’s Dementia

**DOI:** 10.1093/geroni/igaa057.556

**Published:** 2020-12-16

**Authors:** Steve Hwang, Christie Teigland, Zulkarnain Pulungan, Alexis Parente, RoseAnn DePalma

**Affiliations:** 1 Avanir Pharmaceuticals Inc, Aliso Viejo, California, United States; 2 Avalere Health, Washington, District of Columbia, United States

## Abstract

Agitation is common in patients with Alzheimer’s dementia. Lack of a consensus definition has limited our understanding of the prevalence, patient profile, and added healthcare burden of agitation in Alzheimer’s dementia (AAD). We developed an algorithm to identify AAD patients using 100% of Medicare Fee-For-Service administrative claims from 2011-2017. We adapted the International Psychogeriatric Association (IPA) 2015 definition, which had not been tested using real-world data. Patients were required to have 2+ claims ≥30 days apart for Alzheimer’s disease and dementia, and continuous enrollment with medical/pharmacy coverage for 6-months pre- and 12-months post-index diagnosis. The AAD cohort included patients with 2+ claims ≥14 days apart with ICD-9-CM/ICD-10-CM codes selected based on the IPA definition (e.g., dementia with behavioral disturbance, irritability/anger, restlessness/agitation, violent behavior, impulsiveness, wandering). Patients with severe psychiatric disorders were excluded. The final population included 255,669 patients with (34.6%) and 482,710 patients without agitation (65.4%). The mean age in both populations was 82 years. Although the majority of patients in both groups was female, the proportion of males was slightly larger in the AAD group (31.2% vs 29.7%). Patients in the AAD group were also more likely to be low-income (dual-eligible: 44.0% vs 39.6%), disabled (10.4% vs 9.3%), and using antipsychotic and antidepressant medications. The 2 populations had similar comorbidity rates. AAD prevalence may be underestimated using claims data, given imprecise and under-coding. These findings suggest AAD patients can be identified using a claims-based algorithm to support early interventions that can potentially improve outcomes and reduce costs of care.

